# RNA-seq profiling of a radiation resistant and radiation sensitive prostate cancer cell line highlights opposing regulation of DNA repair and targets for radiosensitization

**DOI:** 10.1186/1471-2407-14-808

**Published:** 2014-11-04

**Authors:** Arabella Young, Rachael Berry, Adele F Holloway, Nicholas B Blackburn, Joanne L Dickinson, Marketa Skala, Jessica L Phillips, Kate H Brettingham-Moore

**Affiliations:** School of Medicine, University of Tasmania, Private Bag 23, Hobart, TAS 7000 Australia; QIMR Berghofer Medical Research Institute, Herston, Queensland 4006 Australia; School of Medicine, The University of Queensland, Herston, Queensland 4006 Australia; Menzies Research Institute Tasmania, University of Tasmania, Hobart, Tasmania 7000 Australia; Royal Hobart Hospital, Hobart, Tasmania 7000 Australia

**Keywords:** Radiation, Prostate cancer, RNA-seq, DNA repair, Sensitization

## Abstract

**Background:**

Radiotherapy is a chosen treatment option for prostate cancer patients and while some tumours respond well, up to 50% of patients may experience tumour recurrence. Identification of functionally relevant predictive biomarkers for radioresponse in prostate cancer would enable radioresistant patients to be directed to more appropriate treatment options, avoiding the side-effects of radiotherapy.

**Methods:**

Using an *in vitro* model to screen for novel biomarkers of radioresistance, transcriptome analysis of a radioresistant (PC-3) and radiosensitive (LNCaP) prostate cancer cell line was performed. Following pathway analysis candidate genes were validated using qRT-PCR. The DNA repair pathway in radioresistant PC-3 cells was then targeted for radiation sensitization using the PARP inhibitor, niacinimide.

**Results:**

Opposing regulation of a DNA repair and replication pathway was observed between PC-3 and LNCaP cells from RNA-seq analysis. Candidate genes BRCA1, RAD51, FANCG, MCM7, CDC6 and ORC1 were identified as being significantly differentially regulated post-irradiation*.* qRT-PCR validation confirmed BRCA1, RAD51 and FANCG as being significantly differentially regulated at 24 hours post radiotherapy (*p*-value =0.003, 0.045 and 0.003 respectively). While the radiosensitive LNCaP cells down-regulated BRCA1, FANCG and RAD51, the radioresistant PC-3 cell line up-regulated these candidates to promote cell survival post-radiotherapy and a similar trend was observed for MCM7, CDC6 and ORC1. Inhibition of DNA repair using niacinamide sensitised the radioresistant cells to irradiation, reducing cell survival at 2 Gy from 66% to 44.3% (*p*-value =0.02).

**Conclusions:**

These findings suggest that the DNA repair candidates identified via RNA-seq hold potential as both targets for radiation sensitization and predictive biomarkers in prostate cancer.

**Electronic supplementary material:**

The online version of this article (doi:10.1186/1471-2407-14-808) contains supplementary material, which is available to authorized users.

## Background

Radiation therapy (RT) is commonly used in the treatment of prostate cancer. However, in many cases the survival of cancer cells following RT can result in recurrence and disease progression. Current data indicates that up to 50% of prostate cancer patients undergoing RT experience recurrence of the disease within 5 years of treatment [1, 2]. Regardless of tumour response to RT patients may endure the side-effects, including radiation proctitis, cystitis and erectile dysfunction (reviewed in [3]). A personalised approach to treatment is urgently needed allowing patients unlikely to benefit from conventional RT to be directed towards hypofractionated RT [4] or other therapeutic options.

Understanding the cellular factors contributing to resistance to RT is vital in order to design tests to screen patients prior to receiving therapy and to develop adjuvant treatments to increase tumour cell death. Clinically predictive biomarkers currently in use, for example EGFR testing for treatment with tyrosine kinase inhibitors, rely on the marker being functionally relevant, playing an integral role in therapeutic mechanism. While it has long been known that RT operates by damaging DNA, to date there are no clinically predictive markers available to indicate the likelihood of an effective treatment outcome. It is conceivable that tumours which behave in a similar way in response to RT share similar features which can be used as predictive biomarkers and this hypothesis is currently under study for a range of cancers [5, 6]. Prostate cancer currently lacks predictive biomarkers for treatment response and disease progression which are utilised successfully within other malignancies [7, 8]. Clinicopathologic factors and prostate-specific antigen (PSA) levels currently aid decision making when selecting treatment for the individual patient however there is conflicting evidence as to the predictive and prognostic value of these markers [9–12]. While a number of markers have been identified as prognostic or predictors of recurrence following RT in prostate cancer [13–15] the studies published to date have failed to reach clinical utility and do not consider response to treatment.

In the search for a predictor of response, RNA sequencing (RNA-seq) offers an unbiased screening approach for potential novel biomarkers which relate to RT response. This study compared the post-irradiation transcriptome of a radiation resistant (PC-3) versus radiosensitive prostate cancer cell line (LNCaP). Previous work has demonstrated that these two cell lines have opposing radiosensitivity [16–18] however to date the transcriptome of these cell lines post-irradiation has not been characterised. RNA-seq was used to gain a global perspective of transcriptional changes to investigate the factors integral in response to RT. From the variation in transcriptional activity, specific pathways which relate to differential response were revealed and validated by qRT-PCR. A candidate pathway was selected and targeted for inhibition to determine whether RT sensitisation was possible.

## Methods

### Cell culture

The human prostate cancer LNCaP cell line (ATCC, USA) was cultured in RPMI 1640, while PC-3 cells (ATCC, USA) were grown in Ham’s F-12 K medium. Media was supplemented with 10% FBS and penicillin/streptomycin. For the sensitisation experiments PC-3 cells were treated with 0.1 mM niacinimide (Sigma-Aldrich) for 24 hours prior to and 8 days post-irradiation. This study does not use any human subjects or human material other than continuous cell lines.

### Irradiation set-up

Radiotherapy treatment of prostate cancer cell lines was carried out at the Holman Clinic at the Royal Hobart Hospital, Tasmania, Australia. Irradiation was performed using the Varian® Clinac® 23Ex Linear Accelerator (Varian Medical Systems, Australia) which delivered doses between 2 and 8 Gray (Gy) at 600 monitor units (MU)/min.

### Clonogenic cell survival assays

Prostate cancer cell lines were seeded at 1 × 10^3^ cells/well (PC-3) or 3 × 10^3^ cells/well (LNCaP) and irradiated at 0, 2, 4 or 8 Gy. After 14 days of colony growth, medium was removed and cells washed once in 1 ml of PBS. Colonies were fixed with 700 μL of 3:1 methanol to glacial acetic acid for 5 minutes. Fixative agent was removed and wells air-dried completely prior to staining. Cells were stained for 30 minutes in 500 μL of 1.0% methylene blue (Sigma-Aldrich, USA) in 50% ethanol. Colonies were counted when proliferation from a single viable cell exceeded 50 cells within the colony. Percentage cell survival was determined as the number of colonies post-treatment relative to the number of colonies within the corresponding 0 Gy control.

### RNA isolation

RNA was extracted using TRI reagent® (Sigma-Aldrich, USA). For samples undergoing RNA-seq analysis RNA was subjected to further purification including DNase treatment for 15 minutes at room temperature and a second purification step utilising the RNeasy Plus Micro Kit (Qiagen, USA).

### RNA-seq

RNA integrity was confirmed using the Agilent 2100 Bioanalyser (Agilent Technologies, USA). Next-generation sequencing was performed at the Australian Genome Research Facility (AGRF) using the Illumina Hiseq-2000 RNA-seq sequence production system (50 cycle, single end). Sequences were assessed for quality and then aligned against the human genome using the Tophat aligner (http://tophat.cbcb.umd.edu/). Comparison between the 0, 6 and 24 hour timepoints was performed using Cuffdiff (http://cufflinks.cbcb.umd.edu/).

### Ingenuity pathway analysis

The Ingenuity Pathway Analysis (IPA) program (https://analysis.ingenuity.com/) was utilised to perform a core analysis on the dataset gene files generated by RNA-seq. The gene ID, fold change (>2) and q-value (<0.05) were used for further analysis. Raw data was analyzed using the flexible format and genes identified through human gene symbols in association with the HUGO Gene Nomenclature Committee (HGNC) and Entrez Gene guidelines. Direct and indirect relationships between significant genes were considered.

### Real-time PCR validation

Following irradiation RNA was isolated at specific time-points and reverse transcribed to cDNA using Superscript II reverse transcriptase (Invitrogen, USA). SYBR Green PCR amplification was performed on the Rotor-Gene 2000 real-time cycler (Corbett Research, Australia) using Quantitect SYBR Green PCR mastermix (Qiagen, Germany) according to the manufacturer’s instructions in a total volume of 10 μl, containing 20 ng of cDNA. Cycling conditions were as follows: 95°C for 15 min; 95°C for 15 s, 60°C for 60 s for 35 cycles, followed by melt analysis from 60 to 95°C. Primers are listed in Table 1. Expression levels were normalised to the house-keeping gene GAPDH.

**Table 1:** **Primer sets used in real time PCR**

Primer	Sequence
BRCA1 For	5′-GACAGAGGACAATGGCTTCC-3′
BRCA1 Rev	5′-AGCTCCTGGCACTGGTAGAG-3′
FANCG For	5′-AAGGGGTCACATGAAGATGC-3′
FANCG Rev	5′-GGACGGATCCAGCTCAAATA-3′
RAD51 For	5′-TAGCTCAAGTGGATGGAGCA-3′
RAD51 Rev	5′-TCTGGTTTCCCCTCTTCCTT-3′
MCM7 For	5′-TTTCGCGCCAATTTCGGTTG-3′
MCM7 Rev	5′-ACCTTTTCCTTCTCTAGCGCGT-3′
ORC1 For	5′-CGATTGGCGCGAAGTTTTCT-3′
ORC1 Rev	5′-CTTGTGGGGTAGTGTGCCAT-3′
CDC6 For	5′-CCGTAACCTGTTCTCCTCGT-3′
CDC6 Rev	5′-TAGGTTGTCATCGCCCAGAC-3′
GAPDH For	5′-AAATATGATGACATCAAGAAGGTGGT-3′
GAPDH Rev	5′-AGCCCAGGATGCCCTTGAGGG-3′

### Statistical analysis

Statistical analysis and graph generation was performed in GraphPad Prism version 6.0d for Mac OSX, GraphPad Software, La Jolla California USA, http://www.graphpad.com. The clonogenic assay and gene expression assays were analyzed using repeated measures two-way ANOVA. Cell survival in response to niacinamide exposure was analyzed using repeated measures one-way ANOVA. Cell survival in response to niacinamide and radiation exposure was analyzed using repeated measures two-way ANOVA. The Sídák multiple comparison test was used following each analysis.

### Western blotting

Nuclear extracts were prepared as previously described [19]. Protein concentrations were determined by Bradford Assay (Bio-Rad, USA). Protein extracts were run on a 12% Mini-PROTEAN® TGX™ pre-cast gel (Bio-Rad, USA) and transferred onto nitrocellulose membrane. Western-blot analysis was performed using anti-BRCA1, anti-RAD51 and anti-Sp1 antibodies (Santa Cruz Biotechnology, USA) and the corresponding peroxidase-conjugated secondary antibodies (DAKO, Denmark). Proteins were visualized using the Supersignal West Pico Chemiluminescent kit (Pierce ThermoScientific, USA) according to the manufacturer’s instructions.

## Results

### Clonogenic cell survival post-irradiation demonstrates a significant difference between LNCaP and PC-3 cells

**Figure 1:**
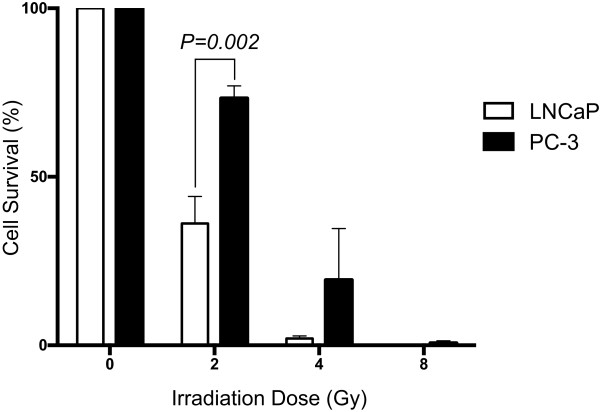
**PC-3 and LNCaP cell survival following irradiation.** Clonogenic assays were carried out to establish difference in cell survival between the two cell lines following radiotherapy treatment at 0, 2, 4 and 8 Gy. Percentage cell survival at each irradiation dose was determined as the proportion of colonies present after treatment (2, 4 and 8 Gy) in comparison to colony numbers within the untreated control sample at 0 Gy. The mean and SEM from three biological replicates are shown, *p*-value determined by two way ANOVA and Sidak’s post test.

LNCaP and PC-3 cells, isolated from prostate cancer lymph and bone metastases respectively, have previously been used as models for radiation sensitivity [17, 18]. In order to confirm these cells behaved as per the literature in our irradiation set-up, the radiation sensitivity of these prostate cancer cell lines was confirmed via clonogenic assay following irradiation. In both cell lines decreased levels of survival were observed with increasing irradiation dose. The PC-3 cell line showed the greatest level of resistance to radiotherapy following 2 Gy irradiation with over 73% cell survival (Figure 1). In contrast, only 36% cell survival was measured for the LNCaP cell line indicating their increased sensitivity (*p*-value 0.002). In both cell lines less than 10% of cells were able to generate viable colonies following 4 Gy irradiation and less than 1% cell survival was observed after 8 Gy irradiation.

### Pathway analysis demonstrates DNA repair and replication were significantly upregulated in the radioresistant cells and downregulated in the radiosensitive cells

To identify differences in genes and pathways affected by irradiation, RNA-seq was performed on the LNCaP and PC-3 cells. Following 2 Gy irradiation (equivalent to the fractionated irradiation dosage commonly received by patients) RNA was isolated at 0, 6 and 24 hours prior to sequencing via the Illumina Hiseq-2000 RNA-seq platform.

**Figure 2:**
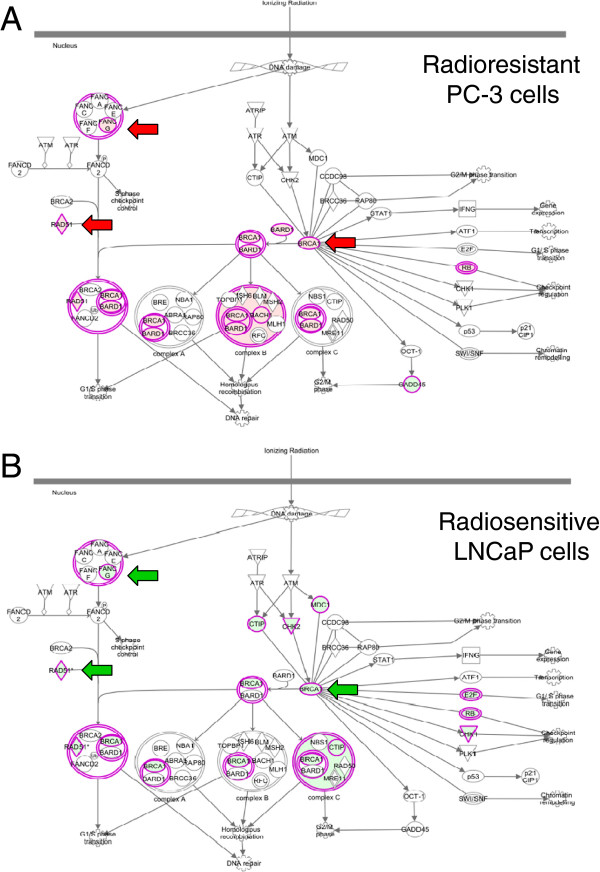
**Pathway analysis highlights opposing regulation of a DNA repair pathway in radioresistant versus radiosensitive cells.** Gene lists determined by RNA-seq for the **(A)** PC-3 and **(B)** LNCaP cell lines 24 hours after 2 Gy irradiation were analysed using IPA. DNA repair pathways were identified as being significantly altered in response to RT (*q*-value 1x10^−10^). Significantly up**-**regulated genes are coloured red and down**-**regulated green, those present within our data set but not significant are shown in grey. Significant genes were defined as reporting a log_2_ fold change >1 and a *q*-value <0.05. Arrows indicate gene products which were found to be oppositely regulated.

The gene lists generated for radiosensitive LNCaP and radioresistant PC-3 cells at 6 and 24 hours following 2 Gy irradiation showed large differences in both the number and type of genes that were transcriptionally activated. Irradiation appeared to impact transcriptional response to a greater extent within the PC-3 cell line with 399 genes significantly differentially regulated by 6 hours (using a 2 fold cut-off). In comparison, at the same time-point only 89 genes were significantly up- or down-regulated for the radiosensitive LNCaP cell line. An unbiased analysis of the gene lists obtained from RNA-seq was then used to uncover pathways involved in radioresponse. Interactions between significantly differentially regulated genes in each cell line were determined using Ingenuity Pathway Analysis (IPA). Two canonical pathways identified were shown to have opposing responses 24 hours after irradiation. The top pathway affected 24 hours post-irradiation for both cell lines was a DNA repair pathway. While key genes within this pathway were significantly up-regulated in the radioresistant PC-3 cell line (Figure 2A) the same subset of genes were oppositely regulated, displaying down-regulation within the radiosensitive LNCaP cell line (Figure 2B). These oppositely regulated genes include BRCA1, RAD51 and FANCG.

The cell cycle control of DNA replication pathway was also observed as the other top canonical pathway affected by irradiation in the cell lines (Additional file 1: Figure S1). Up-regulation of ORC1, CDC6 and the MCM genes was observed at 6 and 24 hours after irradiation in PC-3 cells. In contrast, the LNCaP cell line showed significant down-regulation of the equivalent subset of genes at 24 hours.

Another potential way to find a predictive biomarker is to screen for genes with strong basal expression. The top 10 genes identifiable by RNA-seq were filtered and are listed in Additional file 2: Table S1 along with the contrasting value in the alternate cell line. While these genes may prove to be relevant predictors, a predictive biomarker is more robust when it is functionally relevant. Therefore the focus of this study remained on the genes involved in the two key pathways responsible for differential radiation response.

### BRCA1, RAD51 and FANCG mRNA display significant opposing regulation in response to radiotherapy in the radiosensitive versus radioresistant cells

In order to validate the candidate genes identified by RNA-seq, expression levels following 2 Gy irradiation were assessed using qRT-PCR. The LNCaP cell line demonstrated a slight increase in BRCA1 expression at 6 hours post RT followed by down-regulation within 24 hours post RT to 0.2 fold of the basal level. In contrast BRCA1 mRNA was up-regulated in PC-3 cells post-irradiation to 1.5 fold by 24 hours. Comparison of BRCA1 expression in the LNCaP and PC-3 cell line at 24 hours confirmed opposing regulation with a significant difference observed (*p*-value 0.003).

**Figure 3:**
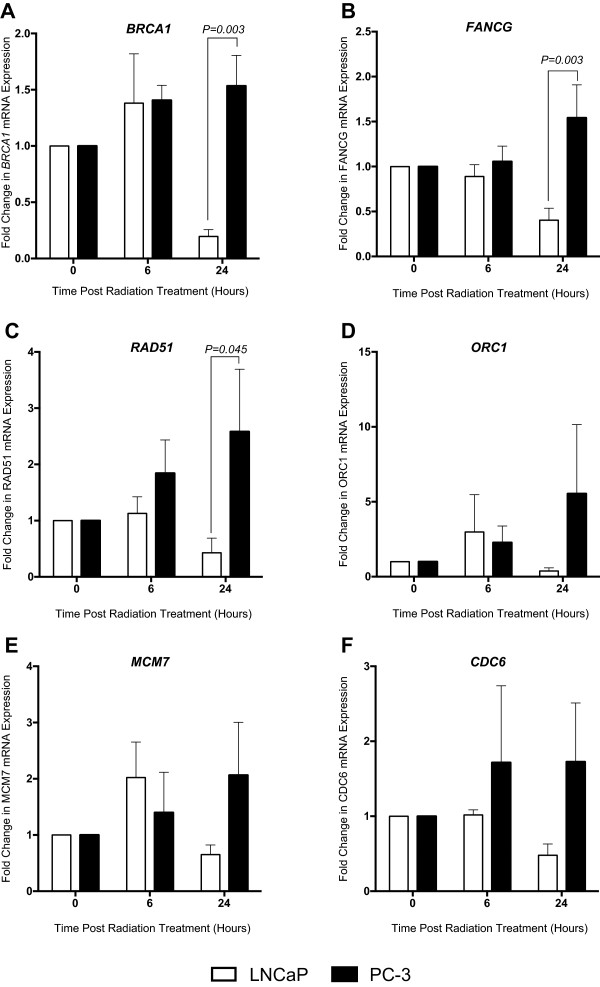
**Regulation of candidate gene expression in response to radiotherapy in LNCaP and PC-3 cell lines.** Cells were exposed to 2 Gy irradiation and RNA was extracted at 0 (non-irradiated) 6 and 24 hours post RT. cDNA was amplified via real-time PCR using primers designed against **(A)** BRCA1, **(B)** FANCG, **(C)** RAD51, **(D)** ORC1, **(E)** MCM7, **(F)** CDC6 and expression levels normalised qRT-PCR to GAPDH. Fold change was calculated relative to the non-irradiated control. Error bars represent standard error of the mean from 3 biological replicates, *p*-values determined by two way ANOVA and Sidak’s post test.

FANCG mRNA also displayed significant opposing regulation at 24 hours post RT being down-regulated by 0.4 fold in the LNCaP cells and up-regulated by 1.5 fold in the PC-3 cells (*p*-value 0.003, Figure 3B).

RAD51 mRNA levels decreased by 0.4 fold basal levels at 24 hours in the LNCaP cells (Figure 3C). In contrast, RAD51 expression was up-regulated in the PC-3 cells by 1.8 and 2.6 fold at 6 and 24 hours post RT respectively. This difference in RAD51 mRNA expression between the two cell lines at 24 hours was shown to be significant (*p*-value 0.045). The DNA replication candidates MCM7, ORC1 and CDC6 mRNA levels displayed a similar trend with down-regulation in the LNCaP cells and up-regulation in the PC-3 cells at 24 hours (Figure 3D-F). However, this difference was found to be non significant.

### Nuclear levels of BRCA1 and RAD51 protein diminished in the radiosensitive cells while increasing in the radioresistant cells post-irradiation

**Figure 4:**
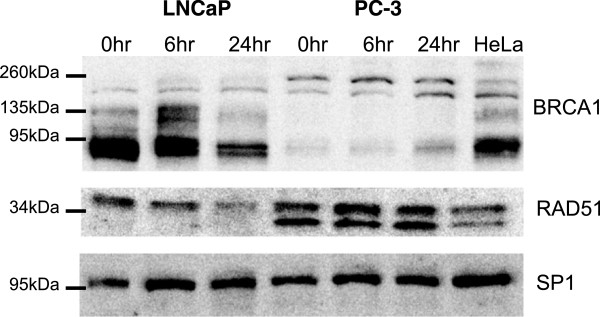
**Nuclear expression of BRCA1 and RAD51 following irradiation in the LNCaP and PC-3 cell lines.** Nuclear proteins were extracted from cells at 0 (non-irradiated), 6 and 24 hours following exposure to 2 Gy irradiation. Protein expression was analysed via Western blot. HeLa nuclear protein extract was utilised as a positive control and membranes were probed for Sp1 as a loading control.

BRCA1 and RAD51 protein expression was then examined as these genes showed significant differential responses following radiation treatment and are directly involved in DNA repair. Western blot analysis was performed on nuclear extracts from non-irradiated and irradiated (2 Gy) cells. Multiple BRCA1 isoforms were detected in the LNCaP cell line at approximately 220 kDa, along with the highly abundant 81 and 85 kDa isoforms (Figure 4). While the levels of full length BRCA1 (220 kDa) remained relatively stable across all time points, the smaller isoforms decreased from 0 to 24 hours post RT. In the PC-3 cell line this lower band was barely detectable at the 0 hour time-point but increased at 24 hours and a similar trend was observed for the full length BRCA1 protein. Another larger band was also detected at 250 kDa being present in the irradiated and non-irradiated PC-3 cells. This band was relatively consistent across all treatment time-points for the PC-3 cells while it was also faintly detected in irradiated LNCaP cells potentially representing phosphorylated BRCA1.

Consistent with the differential transcriptional regulation in the two cell lines (Figure 3C) RAD51 expression decreased following RT in the LNCaP cells with a noticeable reduction in expression at 24 hours. In contrast up-regulation of RAD51 was observed at 6 hours post RT in the PC-3 cell line, and appeared to remain greater than the basal level at 24 hours post RT. The detection of a strong, second band just below 34 kDa was observed in the PC-3 cells at all time points. This second band was confirmed to be a novel non-functional RAD51 splice variant (Additional file 3 and Additional file 4: Figure S2). The amplicon lacked the sequence corresponding to exon 9 of RAD51, a previously identified sequence variant [20] however the variant sequenced from the PC-3 cells was also missing 130 bp of the 3 prime end of exon 8. The predicted amino acid sequence of the variant consists of codons 1 to 214 and 299 to 339 of full length RAD51. The translated protein contains the Walker A ATP binding motif, but is lacking the Walker B ATP binding motif.

### The PARP inhibitor niacinamide successfully sensitised radioresistant prostate cancer cells to irradiation

**Figure 5:**
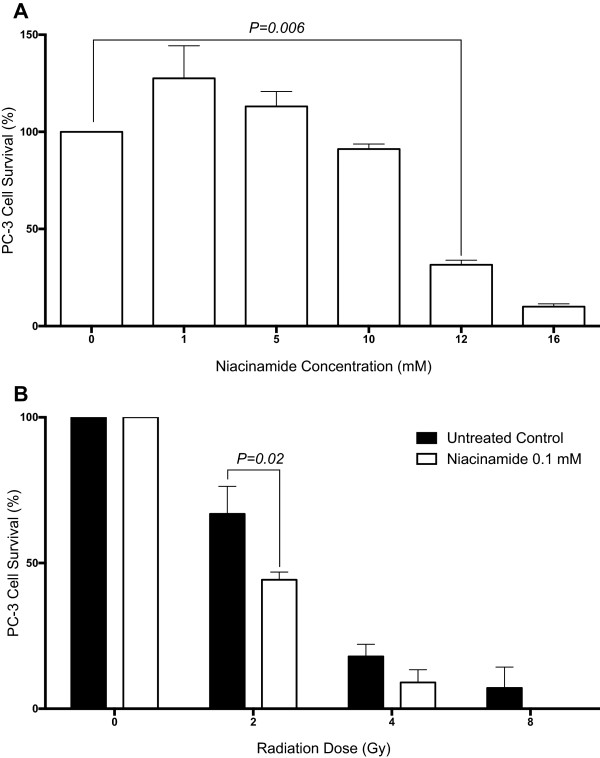
**Adjuvant treatment with niacinamide significantly increases the radiation response of PC-3 cells. A)** PC3 cells were incubated with varying concentrations of the PARP inhibitor niacinamide for 8 days and cell colonies of greater than 50 cells were counted. Cell survival percentages were calculated relative to the number of colonies counted in the untreated population. Error bars represent standard error of the mean from 3 biological replicates. *p*-values determined by one way ANOVA. **B)** PC-3 cells were incubated with or without niacinamide for 24 hours prior to and 8 days following exposure to 0, 2, 4 or 8 Gy irradiation. Following incubation, colonies of greater than 50 cells were counted, and cell survival percentages were calculated relative to the appropriate non-irradiated colony counts. Error bars represent standard error of the mean from 3 biological replicates. *p*-values as determined by two way ANOVA and Sidak’s post test.

Poly ADP-ribose polymerase (PARP) inhibitors have previously been shown to inhibit DNA repair and down-regulate BRCA1 and RAD51 [21]. To determine whether radioresistant cells could be rendered sensitive by targeting the DNA repair pathway, the PARP inhibitor niacinamide was added to cells prior to irradiation. Prior to sensitisation assays the toxicity of niacinamide was determined and the optimal concentration selected as a low and clinically relevant dosage without a significant effect on cell survival (Figure 5A).

Cells were treated with 0.1 mM niacinimide for 24 hours prior to irradiation and 8 days post-irradiation. Cells were irradiated at 0, 2, 4 and 8 Gy and survival assessed via clonogenic assay. As shown in Figure 5B, for untreated PC-3 cells approximately 66% cell survival was measured following 2 Gy irradiation. Treatment of cells with niacinamide significantly reduced cell survival to 44.3% (*p*-value 0.02). This result was reiterated at 4 Gy with 18% of untreated cells surviving in contrast to cells sensitised with niacinamide exhibiting only 9% cell survival (Figure 5B). Following 8 Gy irradiation in untreated cells, 7% cell survival was observed while in contrast no formation of viable colonies within the niacinamide-treated PC-3 cells was apparent.

## Discussion

The genetic heterogeneity observed in prostate cancer results in tumours from different individuals displaying significant variation in response to treatment [22, 23]. Understanding the molecular pathology that contributes to this variation will enable tailoring treatment to specific tumour subtypes [24, 25]. In order to gain insight into the molecular mechanism underpinning radiation sensitivity an unbiased approach was employed to identify differential gene expression relating to radioresponse. It has long been known that ionising radiation induces several forms of DNA damage [26, 27] therefore, it was not surprising that the two most significant pathways observed to be altered following irradiation relate functionally to DNA repair and replication. In a previously unreported finding, these pathways were shown to be oppositely regulated in the radioresistant PC-3 cell line versus the radiosensitive LNCaP cell line. The radioresistant cells, by actively up-regulating genes in these pathways promoted cell survival. In contrast the radiosensitive cells inhibit expression of these very same genes, leading to cell death. The time taken to initiate DNA repair following irradiation has been previously identified as a major factor in determining radioresponse [28–30]. Identifying the level of response of these genes during the initial acute phase following irradiation may indicate the level of radiosensitivity.

The regulation of DNA repair and replication genes BRCA1, RAD51, FANCG, ORC1, CDC6 and MCM7 correlated with radiation sensitivity in the LNCaP and PC-3 cell lines. While BRCA1, FANCG and RAD51 have previously been linked to various treatment responses in a number of cancers [31–33] limited research exists investigating the association between RT-induced regulation of these genes in radiation resistant prostate cancer. These genes appear to be key in regulating radiation response and therefore these pathways or their upstream regulators may prove to be a good predictor of treatment outcome. The DNA replication genes identified via RNA-seq warrant further investigation, while a trend was confirmed by qRT-PCR this was not significant. Future work in further characterising several of the candidates in patient biopsies is needed. With further investigation variants or basal patterns may prove to be predictive of response.

BRCA1 expression was found to have a strong association with radiation response, with significant opposing regulation observed between the two cell lines. BRCA1 has been identified as a primary regulator of the repair of DNA double- stranded breaks (DSB), which are formed on exposure to ionising radiation (reviewed in [34]). BRCA1 mutation and exogenous down-regulation has also previously been found to increase sensitivity to RT in a variety of cancer cell lines [35, 36] however no evidence is available for prostate cancer cells. The novel finding that prostate cancer cells with disparate radiosensitivity exhibit opposing regulation of BRCA1 following RT supports its involvement in determining radiation response. Interestingly, the initial RNA-seq analysis demonstrated that a key transcription factor in BRCA1 regulation, *E2F1* [37, 38] was significantly down-regulated in the LNCaP cell line at 24 hours post-RT, which may explain the decrease in BRCA1 expression at the same time point. This transcription factor is also involved in regulating RAD51 and CDC6 expression [39, 40]. In terms of potential implications for this finding it is unlikely that basal BRCA1 mRNA levels could be used as a predictive biomarker. While post RT levels of BRCA1 are more informative, it is unlikely patient biopsies could be taken at this point. Regardless, BRCA1 is an appealing target for sensitisation and screening BRCA1 mutations may help direct patients to their most optimal treatment.

It should also be noted that, as is the case in patient tumours, the LNCaP and PC-3 cell lines have many inherent differences. For example LNCaP cells are androgen sensitive and p53 positive [41, 42] while the PC-3 cells are androgen resistant and p53 null [42, 43]. Regardless, p53 was not identified as being significantly differentially regulated in the p53 positive cell line post RT. Previous research has investigated p53 status as a predictive biomarker for radiation response in prostate cancer however, the large range of possible p53 mutations has led to conflicting results. Whilst some studies found that resistant tumours had a higher level of expression [44, 45] others concluded that p53 expression was comparable in both radiation sensitive and radiation resistant tumours [46, 47]. Therefore, investigations into p53 targets, such as BRCA1 and RAD51, as predictors may be a more promising avenue for future research.

Radiation sensitisation strategies have been successful in the treatment of a variety of cancers [48–51] however clinically effective strategies have remained elusive in prostate cancer. This study demonstrated the efficacy of PARP inhibition in sensitising the radiation resistant PC-3 prostate cancer cell line to the effects of RT. Niacinamide is a known inhibitor of PARPs which are involved in the repair of single-stranded breaks (SSB) [52]. All PARP inhibitors are based on the structure of niacinamide and use the same competitive binding strategy [53]. Importantly, PARP inhibition has also previously been demonstrated to down-regulate BRCA1 and RAD51 [21]. Much of the current research surrounds the use of PARP inhibitors as effective mono-therapy for breast and ovarian cancers with BRCA1 and BRCA2 mutations [54–56]. However, evidence for their radiation sensitisation capabilities is emerging from *in vivo* studies [57]. The use of niacinamide as a sensitisation strategy is an appealing possibility, due to the fact this compound has FDA approval and has beneficial effects (reviewed in [58]).

RAD51 is another promising target to enhance response to RT as clinically approved inhibitors are already available. RAD51 has previously been proposed as a possible target for radiosensitisation through inhibition using imatinib in prostate cancer xenografts [59]. Recent evidence demonstrates that imatinib down-regulates RAD51 expression and sensitises bladder and glioma cancer cells to RT [60, 61]. Further molecular characterisation of the precise involvement of BRCA1 and RAD51 may contribute to more targeted radiosensitisation strategies.

## Conclusions

This study is the first to characterise the post irradiation transcriptome of two prostate cancer cell lines with divergent responses to RT commonly used in research. RNA-seq analysis revealed the potential for BRCA1 and RAD51 as biomarkers for radiation response. RT-induced regulation of both transcription and nuclear protein localisation was found to be associated with the differential radiation response of LNCaP and PC-3 prostate cancer cell lines. Given the role of BRCA1 and RAD51 in the homologous repair of DSBs, it is likely that their increased expression contributes to the repair of the DNA damage caused by RT to promote survival in resistant cells. In addition, PARP has been identified as a putative target for adjuvant sensitisation strategies.

Translational research has an overall aim to be used clinically, providing benefits for patients, therefore the ability to validate *in vitro* based markers *in vivo* will be essential. Analysis of the behaviour of prostate cancer cell lines provides a reference point for possible traits that cause RT resistance. Importantly, the data generated by RNA-seq has provided potential leads on influential pathways, which are affected by irradiation. In addition, inhibition of gene products from these pathways can be used to sensitise prostate cancer cells to cell death following RT. Additional validation of these targets using patient biopsies will be imperative to understanding their potential clinical utility. Similarly, sensitisation agents require validation in mouse models (such as TRAMP and PTEN-induced prostate-specific cancer formation PTENfl/fl; probasin-Cre mice) prior to determining their suitability for clinical trials. Proving candidate markers and sensitisation agents to be clinically significant remains a definite challenge. However, with improving technology to recognise molecular subtleties which separate particular treatment responses, new opportunities for tailoring therapeutics will become available. This will enable increased translational research into the individualised management of prostate cancer patients providing advantages to the overall survival benefit received by patients. As niacinamide is a safe, well tolerated FDA approved vitamin supplement, its sensitisation effects could also be investigated by surveying patients taking such supplements followed by correlation with response data. Finally, the biomarkers and sensitisation strategy identified in this study may not only prove to be effective in prostate tumours, but may be relevant to numerous cancer types as a mechanism for inherent radiation resistance.

## Electronic supplementary material

Additional file 1: Figure S1: Differential regulation of cell cycle control of chromosomal replication pathway in PC-3 and LNCaP cells. IPA was performed on gene lists generated by RNA-seq of the A) PC-3 and B) LNCaP cell lines 24 hours following 2 Gy irradiation. The DNA replication pathway was identified as being significantly altered in response to RT (*q*-value 5x10^−8^). Significantly up-regulated genes are coloured red and down-regulated green, genes that showed differential expression at non-significant levels are shown in grey. Significant genes were defined as reporting a log_2_ fold change >1 and a *q*-value <0.05. (PDF 191 KB)

Additional file 2: Table S1: Top 10 known genes with highest basal expression in the LNCaP versus PC-3 cells. (XLSX 10 KB)

Additional file 3: **Additional methods.** (DOC 22 KB)

Additional file 4: Figure S2: Alignment of the amino acid sequences of RAD51 and a novel RAD51 variant. PCR was performed utilising primers designed to amplify full length *RAD51* and a previously identified variant, *RAD51∆ ex9.* The smaller PCR product was extracted from the gel and sequenced. The resultant sequence was translated into the predicted amino acid sequence of the amplicon and aligned with the amino acid sequence of full length RAD51. Grey shading represents amino acids that are missing from the novel variant. Arrows indicate exons 8, 9 and 10. The Walker A ATP binding motif is indicated by the black box, whilst the Walker B ATP binding motif is indicated in bold type and underlined. *represents a stop codon. (PDF 63 KB)

## Abbreviations

DSB: Double stranded break
Gy: Gray
IPA: Ingenuity pathway analysis
PARP: Poly ADP ribose polymerase
qRT-PCR: Quantitative reverse transcription polymerase chain reaction
RNA-seq: RNA sequencing
RT: Radiation therapy
SSB: Single stranded break.
