# Supplementary figures and images for: RNA-seq profiling of a radiation resistant and radiation sensitive prostate cancer cell line highlights opposing regulation of DNA repair and targets for radiosensitization
Source: BMC Cancer. 2014 Nov 4;14:808. doi: 10.1186/1471-2407-14-808 (PMC4233036; doi:10.1186/1471-2407-14-808)

A

Nucleus

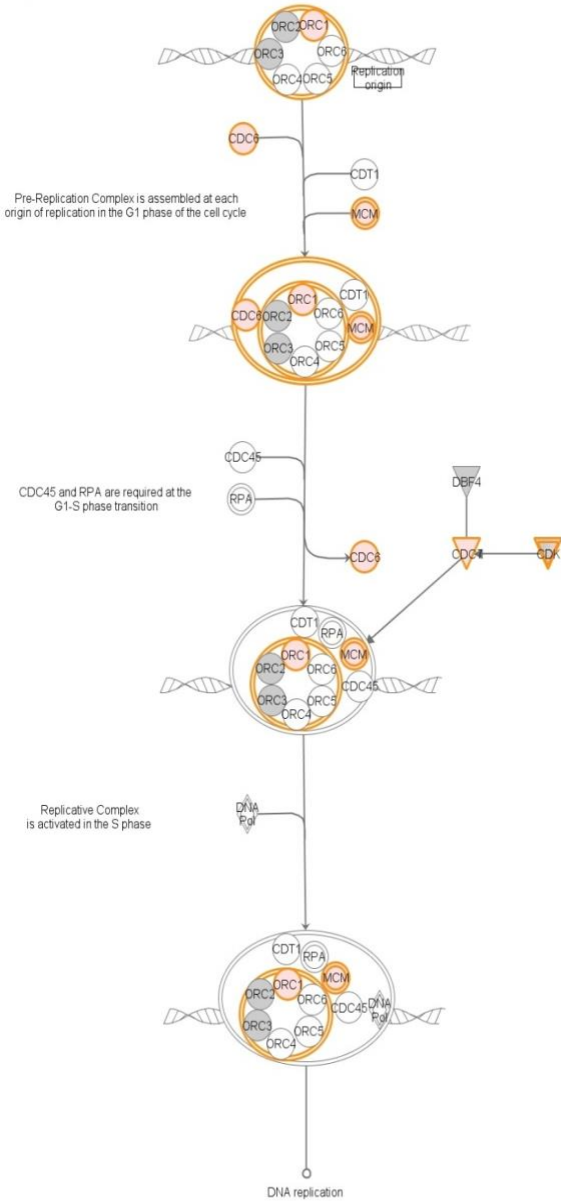

B

Nucleus

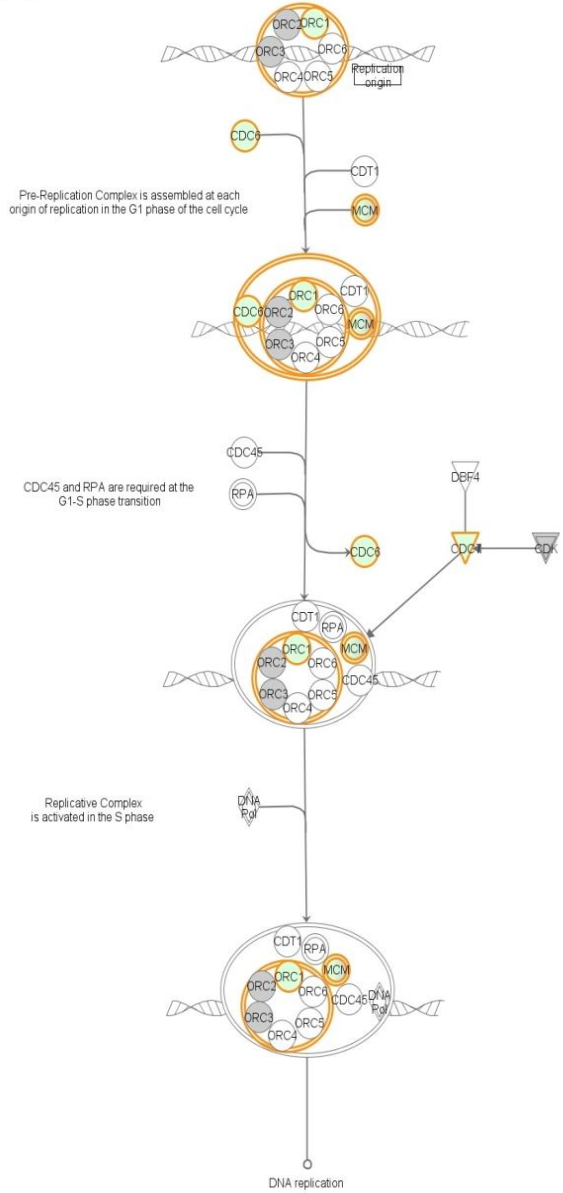

Supplement: Supplementary file 1 — Additional file 1: Figure S1: Differential regulation of cell cycle control of chromosomal replication pathway in PC-3 and LNCaP cells. IPA was performed on gene lists generated by RNA-seq of the A) PC-3 and B) LNCaP cell lines 24 hours following 2 Gy irradiation. The DNA replication pathway was identified as being significantly altered in response to RT (q-value 5x10^−8^). Significantly up-regulated genes are coloured red and down-regulated green, genes that showed differential expression at non-significant levels are shown in grey. Significant genes were defined as reporting a log2 fold change >1 and a q-value <0.05. (PDF 191 KB) [file 12885_2014_4994_MOESM1_ESM.pdf]
