# Supplementary material for: RNA-seq profiling of a radiation resistant and radiation sensitive prostate cancer cell line highlights opposing regulation of DNA repair and targets for radiosensitization
Source: BMC Cancer. 2014 Nov 4;14:808. doi: 10.1186/1471-2407-14-808 (PMC4233036; doi:10.1186/1471-2407-14-808)
Supplement: Supplementary file 3 — Additional file 3: Additional methods. (DOC 22 KB) [file 12885_2014_4994_MOESM3_ESM.doc]

**Additional methods**

RAD51 sequence variant

RNA isolated from PC-3 cells was reverse transcribed and amplified using the RAD51 Δexon 9 primers (For:TTTGGAGAATTCCGAACTGG, Rev: AGGAAGACAGGGAGAGTCG). PCR products were electrophoresed and bands excised and purified using the GFX PCR DNA and Gel Band Purification Kit (GE Healthcare, USA). Sequencing was then performed using the Veriti Thermocycler (Applied Biosystems, USA). Sequence data was compared to *Homo sapiens* RefSeq RNA using BLAST (<http://blast.ncbi.nlm.nih.gov/Blast.cgi?CMD=Web&PAGE_TYPE=BlastHome>) with the discontiguous megablast parameter selected.
