# Supplementary material for: RNA-seq profiling of a radiation resistant and radiation sensitive prostate cancer cell line highlights opposing regulation of DNA repair and targets for radiosensitization
Source: BMC Cancer. 2014 Nov 4;14:808. doi: 10.1186/1471-2407-14-808 (PMC4233036; doi:10.1186/1471-2407-14-808)
Supplement: Supplementary file 4 — Additional file 4: Figure S2: Alignment of the amino acid sequences of RAD51 and a novel RAD51 variant. PCR was performed utilising primers designed to amplify full length RAD51 and a previously identified variant, RAD51∆ ex9. The smaller PCR product was extracted from the gel and sequenced. The resultant sequence was translated into the predicted amino acid sequence of the amplicon and aligned with the amino acid sequence of full length RAD51. Grey shading represents amino acids that are missing from the novel variant. Arrows indicate exons 8, 9 and 10. The Walker A ATP binding motif is indicated by the black box, whilst the Walker B ATP binding motif is indicated in bold type and underlined. *represents a stop codon. (PDF 63 KB) [file 12885_2014_4994_MOESM4_ESM.pdf]

```
RAD51           1    MAMQMQLEANADTSVEEESFGPQPISRLEQCGINANDVKKLEEAGFHTVEA   50
                     ||||||||||||||||||||||||||||||||||||||||||||||||||
Novel variant   1    MAMQMQLEANADTSVEEESFGPQPISRLEQCGINANDVKKLEEAGFHTVEA   50

RAD51           51   VAYAPKKEL I N I KGISEAKADK I LAEAAKLVPMGFTTATEFHQRRSE I I Q   100
                     ||||||||||||||||||||||||||||||||||||||||||||||||||
Novel variant   51   VAYAPKKEL I N I KGISEAKADK I LAEAAKLVPMGFTTATEFHQRRSE I I Q   100

RAD51           101  I TTGSKELDKLLQGG I ETGS I TEMFGEFRTGKTQ I CHTLAVTCQLP I KRG   150
                     ||||||||||||||||||||||||||||||||||||||||||||||||||
Novel variant   101  I TTGSKELDKLLQGG I ETGS I TEMFGEFRTGKTQ I CHTLAVTCQLP I KRG   150

RAD51           151  GGEGKAMYIDTEGTFRPERLLAVAERYGLSGSDVLDNVAYARAFNTDHQT   200
                     ||||||||||||||||||||||||||||||||||||||||||||||||||
Novel variant   151  GGEGKAMYIDTEGTFRPERLLAVAERYGLSGSDVLDNVAYARAFNTDHQT   200

                                    <----------- Exon 8 ----------
RAD51           201  QLLYQASAMMVESRYALLIVDSATALYRTDYSGRGELSARQMHLARFLRM   250
                     ||||||||||||||
Novel variant   201  QLLYQASAMMVESR ------------------------------------   250

                     ---->  <----------- Exon 9 ------------------->  <--
RAD51           251  LLRLADEFGVAVVITNQVVAQVDGAAMFAADPKKPIGGNIIAHASTTRLY   300
                                                                     ||
Novel variant   215  ------------------------------------------------ LY   216

                     ------------- Exon 10 ----------->
RAD51           301  LRKGRGETRICKIYDSPCLPEAEAMFAINADGVGDAKD*               339
                     ||||||||||||||||||||||||||||||||||||||
Novel variant   217  LRKGRGETRICKIYDSPCLPEAEAMFAINADGVGDAKD*               254
```
